# *Notes from the Field:* Underreporting of Maternal Hepatitis C Virus Infection Status and the Need for Infant Testing — Oregon, 2015

**DOI:** 10.15585/mmwr.mm6706a6

**Published:** 2018-02-16

**Authors:** Stephanie D. Snodgrass, Tasha M. Poissant, Ann R. Thomas

**Affiliations:** ^1^Department of Infectious Diseases and Microbiology, Graduate School of Public Health, University of Pittsburgh, Pennsylvania; ^2^Acute and Communicable Disease Prevention, Public Health Division, Oregon Health Authority.

The rate of deliveries to women with hepatitis C virus (HCV) infection has increased sharply in the United States (*1*). A review of 2009–2014 birth certificate data from 47 states that report HCV status of the mother on infant birth certificates found an 89% increase in prevalence of maternal HCV infection, from 1.8 per 1,000 live births in 2009 to 3.4 in 2014 (*2*). During the same period in Oregon, the prevalence of births to women with HCV infection increased 33%, from 2.91 per 1,000 live births in 2009 to 3.87 in 2014. Although North American Society for Pediatric Gastroenterology, Hepatology, and Nutrition guidelines recommending testing of infants born to HCV-infected mothers at age 18 months were published in 2012 (*3*), a recent study conducted by the Philadelphia Department of Health found that only 16% of infants born to HCV-infected mothers in Philadelphia during 2011–2013 had been appropriately tested (*4*). To evaluate the completeness of reporting of maternal HCV infection in the state, the Oregon Health Authority compared birth certificate data with data reported to the state’s Acute and Communicable Disease Prevention (ACDP) program. The results of that comparison suggested that use of birth certificate data to identify infants born to women with HCV infection underestimates the prevalence of maternal HCV infection and that the majority of exposed infants did not receive age-appropriate HCV testing.

The following two data sources were used in the Oregon analysis: 1) female HCV cases reported to ACDP during 2001–2015 and 2) Oregon birth certificate records from all live births in 2015, which included maternal HCV status. Using a probabilistic record linkage program for registry database linkage, ACDP surveillance records for HCV-positive women aged 15–50 years in 2015 were matched with mothers’ names and dates of birth from all live births in Oregon. The study was considered public health practice under Oregon statute and did not require review by the Oregon Health Authority’s public health institutional review board.

Among women with a positive HCV laboratory result reported to ACDP during 2001–2015, a total of 13,058 were aged 15–50 years in 2015. Among 44,712 women who had a live birth in 2015, maternal HCV infection was recorded on the birth certificates of 181 (0.4%) infants. Among these women, 150 (82.9%) were matched by name and date of birth to women with a positive HCV laboratory result reported to ACDP; 31 (17.1%) of the 181 women identified in birth certificates had not been reported to ACDP during the period 2001–2015 (Table). An additional 113 women with a positive HCV laboratory result reported to ACDP matched women who had a live birth during 2015 but did not have a diagnosis of maternal HCV infection recorded on the birth certificate. Thus, the linkage resulted in identification of 294 women with HCV infection who gave birth in 2015, a 62% increase over the estimate of 181 women using birth certificates alone.

**TABLE:** Comparison of women identified with hepatitis C virus (HCV) infection on infant’s birth certificates in 2015 with female HCV cases reported to the ACDP program — Oregon, 2001–2015

Laboratory diagnosis of HCV infection reported to ACDP	Maternal HCV infection recorded on infant’s birth certificate		
	Yes	No	Total
**Yes**	150	113	263
**No**	31	—	31
**Total**	**181**	**113**	**294**

**Abbreviation:** ACDP = Acute and Communicable Disease Prevention.

Assuming an estimated 5.8% rate of perinatal HCV transmission (*5*), 17 of the 294 exposed infants would be expected to have acquired HCV infection. As of July 31, 2017 (at which time all children born in 2015 would have reached age 18 months, the recommended age for testing children born to HCV-infected mothers), the ACDP database had recorded five positive HCV reports from infants born in 2015. Negative HCV tests are not reportable in Oregon, so it is unknown how many of the exposed infants were appropriately tested. However, the discrepancy between the number of reported positive results and the expected number based on estimates of perinatal transmission suggests that as many as 12 infants with HCV might not have been tested by age 18 months.

This investigation suggests that the use of birth certificate data to identify infants born to women with HCV infection underestimates the prevalence of maternal HCV infection and that the majority of exposed infants do not receive age-appropriate HCV testing. New public health strategies are needed to actively identify infants at risk for HCV infection and ensure that they are tested appropriately.
